# Updated Guidelines for the Diagnosis and Treatment of Endometrial Carcinoma: The Polish Society of Gynecological Oncology (2025v)

**DOI:** 10.3390/curroncol32060340

**Published:** 2025-06-09

**Authors:** Jacek J. Sznurkowski, Lubomir Bodnar, Anna Dańska-Bidzińska, Andrzej Marszałek, Pawel Blecharz, Anita Chudecka-Głaz, Dagmara Klasa-Mazurkiewicz, Artur Kowalik, Agnieszka Zołciak-Siwinska, Aleksandra Zielinska, Mariusz Bidziński, Włodzimierz Sawicki

**Affiliations:** 1 Profesor Sznurkowski Podmiot Leczniczy, ul. Stefana Żeromskiego 23A, 81-246 Gdynia, Poland; 2 Faculty of Medical and Health Sciences, University in Siedlce, 08-110 Siedlce, Poland; lubomirbodnar.lb@gmail.com; 3 Department of Gynaecological Oncology, Maria Sklodowska-Curie National Research Institute of Oncology, 00-001 Warsaw, Poland; ad.bidzinska@gmail.com (A.D.-B.); bidzinski.m@gmail.com (M.B.); 4 Department of Tumor Pathology and Prophylaxis, Poznan University of Medical Sciences, 60-806 Poznan, Poland; andrzej.marszalek@wco.pl; 5 Department of Tumor Pathology, Greater Poland Cancer Center in Poznan, 61-866 Poznan, Poland; 6 Department of Gynaecologic Oncology, Maria Sklodowska-Curie National Research Institute of Oncology, Krakow Branch, 00-001 Warsaw, Poland; pblecharz@gmail.com; 7 Department of Gynecological Surgery and Gynecological Oncology of Adults and Adolescents, Pomeranian Medical University, 70-111 Szczecin, Poland; anita.chudeckaglaz@gmail.com; 8 Department of Gynecology and Obstetrics, Medical University of Gdańsk, 80-214 Gdańsk, Poland; dagmara.klasa-mazurkiewicz@gumed.edu.pl; 9 Department of Molecular Diagnostics, Holy Cross Cancer Center, 25-735 Kielce, Poland; artur.kowalik@onkol.kielce.pl; 10 Division of Medical Biology, Institute of Biology, Jan Kochanowski University, 25-406 Kielce, Poland; 11 Department of Radiotherapy, Maria Sklodowska-Curie National Research Institute of Oncology, 00-001 Warsaw, Poland; agnieszka.zolciak@wp.pl; 12 Department of Obstetrics and Gynecological Oncology, Medical University of Warsaw, 00-575 Warsaw, Poland; ola@adelogic.pl (A.Z.); saw55@wp.pl (W.S.)

**Keywords:** endometrial cancer, diagnosis, guidelines, AGREE, POLE, MMRD, NSMP, TP53abn, I/O, immunotherapy, adjuvant, systemic, update, targeted therapy

## Abstract

In 2023, the Polish Society of Gynecologic Oncology (PSGO) published clinical recommendations for the diagnosis, treatment, and care of women with endometrial cancer (EC), developed using the AGREE II (Appraisal of Guidelines for Research and Evaluation) tool. A 2025 update was initiated in response to new evidence, particularly regarding systemic therapies for metastatic, advanced, or recurrent EC, and the introduction of an updated FIGO classification. A targeted literature review identified relevant phase III clinical trials and systematic reviews, including RUBY, GY-018, AtTend, and DUO-E. These trials were critically assessed by an Expert Panel in accordance with the AGREE II methodology. Updated recommendations were formulated based on this evidence, with a comparative analysis of the old and new FIGO staging systems and visual updates to treatment pathways. Key changes include the addition of immunotherapy (I/O) plus chemotherapy (CHTH) as first-line treatment for all molecular subtypes of high-grade endometrioid and non-endometrioid carcinomas, replacing chemotherapy alone. For MMRp-positive cases, the 2025 version introduces the use of Olaparib alongside Durvalumab and CHTH. HER2-positive MMRp serous carcinoma remains eligible for trastuzumab in combination with CHTH. Second-line treatment guidance remains unchanged for patients who did not receive I/O plus CHTH initially. However, options for those previously treated with this combination are still under evaluation. This update ensures alignment with the latest international standards and reinforces evidence-based, personalized care for EC patients.

## 1. Background and Methodology

**Figure 1:**
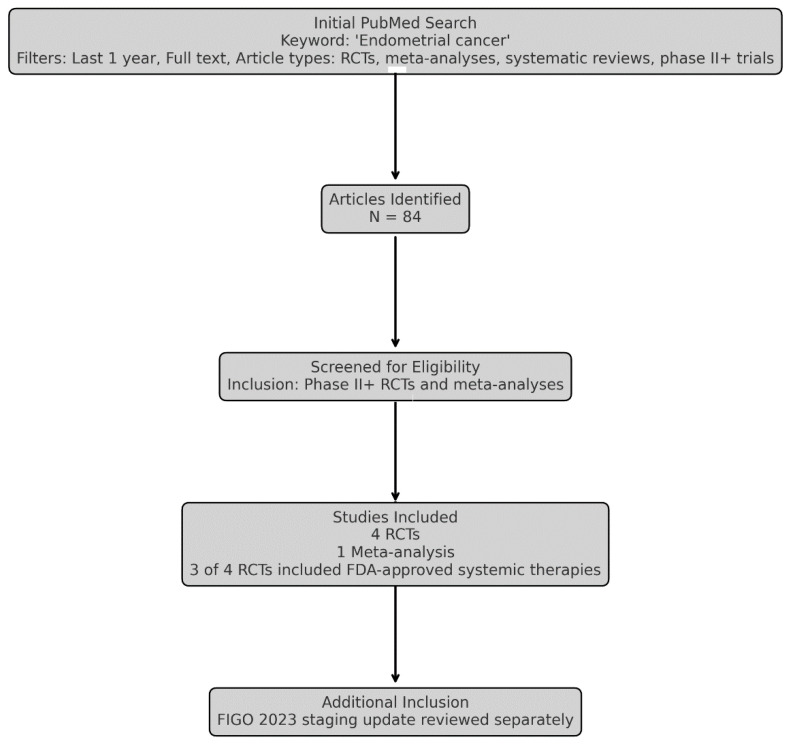
Flow diagram summarizing the study selection process for guideline update.

The PTGO Recommendations Updates provide targeted revisions to selected guideline recommendations in response to new, practice-changing evidence. A structured PubMed search identified four relevant randomized controlled trials (RCTs) and one meta-analysis of RCTs (Figure 1).

In addition, the FIGO 2023 staging update was reviewed separately, given its emerging and not yet fully defined role in the current management of endometrial cancer. These updates are grounded in a thorough evidence review and adhere to the rigorous guideline development processes detailed in the PTGO Guideline Methodology Manual (AGREE II) [1]. In summary, AGREE II is a structured tool designed to ensure the quality, clarity, and applicability of clinical guidelines. PSGO followed a six-step process that included forming a multidisciplinary team, gathering and evaluating scientific evidence, aligning with international standards (ESGO/NCCN), and incorporating external expert reviews. The evidence was graded based on a hierarchy established by the Polish Agency for Health Technology Assessment and Tariff System (AOTMiT), ranging from randomized controlled trials (highest) to expert opinion (lowest). Recommendations were categorized by both evidence strength and expert consensus, ensuring a transparent and rigorous classification system [1]. The primary objective of this concise review is to deliver updated recommendations promptly, ensuring that healthcare practitioners and the public have access to the most current and reliable cancer care guidance.

## 2. Key Findings

### 2.1. Comparison of FIGO 2023 and 2009

The 2023 update to the International Federation of Gynecology and Obstetrics (FIGO) staging system for endometrial and gynecological carcinomas introduces significant changes. It now incorporates factors such as tumor type, grade, lymphovascular space invasion, and molecular alterations, moving beyond the purely anatomical criteria established in the FIGO 2009 edition [2,3]. This novel approach has faced criticism, with some experts advocating for a comprehensive evaluation across multiple institutions and global stakeholder engagement before implementing such changes to ensure their full impact is understood and widely supported [4,5].

Anatomic staging continues to be more universally applicable, as it aligns effectively with established regional and national guidelines, such as those provided by ESGO/ESTRO/ESP and NCCN, which consider local resources and risk factors. Nevertheless, notable differences exist between the 2023 FIGO staging system and the 2021 ESGO/ESTRO/ESP guidelines. One key distinction is that ESGO/ESTRO/ESP differentiates high-grade (FIGO grade 3) endometrioid carcinoma from non-endometrioid carcinomas, whereas the 2023 FIGO classification consolidates both under the category of ‘aggressive histological types’. Additionally, while both frameworks incorporate POLEmut and p53abn molecular groups in risk and stage assessments, the same does not hold for mismatch repair (MMR) deficient and no specific molecular profile (NSMP) groups. ESGO/ESTRO/ESP utilizes these molecular groups to stratify tumors into intermediate, intermediate-high, and high-risk categories, whereas the 2023 FIGO system does not include them.

While a universal system for risk assessment is ideal, it requires uniform diagnostic and therapeutic resources worldwide—a condition unlikely to be met soon. Thus, a universal approach like FIGO 2023 may exacerbate disparities in cancer care between resource-rich and resource-poor settings, moving away from the goal of personalized medicine [4]. The difference between FIGO 2009 and FIGO 2023 staging systems are presented in Table 1.

#### Expert Opinion

The FIGO 2009 staging system should remain the preferred choice for pre-treatment staging, as it relies solely on anatomical parameters readily accessible through imaging and clinical evaluation. This system provides a straightforward, pre-intervention radiological and clinical stage, which has been the foundation of clinical trials, supporting decision-making based solely on anatomical factors.

The FIGO 2023 staging system introduces additional non-anatomical parameters, including tumor type, grade, lymphovascular space invasion, and molecular alterations, offering a more personalized staging approach with potential relevance for prognosis and management. However, these parameters are generally available only after surgery, limiting the FIGO 2023 system’s utility for pre-treatment planning, especially in early-stage disease (see impact of LVSI).

The FIGO 2023 staging system’s role in planning adjuvant therapy remains uncertain, as no randomized controlled trials (RCTs) have yet incorporated it. Given this limitation, expert opinion currently advises against rearranging risk groups for adjuvant treatment based on the new FIGO 2023 risk stages until tested in RCTs [Expert opinion] [strength of evidence of V].

### 2.2. Newly Published RCTs & Meta-Analysis

#### 2.2.1. RUBI (antyPD1)

The RUBY trial enrolled 494 patients with a median follow-up of 25 months.

Patients were randomly assigned in a 1:1 ratio to receive either dostarlimab (500 mg) or placebo, alongside carboplatin (AUC 5 mg/mL/min) and paclitaxel (175 mg/m^2^) every 3 weeks for the first six cycles. Following this, dostarlimab (1000 mg) or placebo was administered every 6 weeks for up to 3 years.

Mismatch repair (MMR) status and microsatellite instability (MSI) testing were primarily conducted at local laboratories. Central testing was performed in cases where local results were unavailable. Accepted methods for local assessment included immunohistochemistry (IHC), next-generation sequencing (NGS), and polymerase chain reaction (PCR) assays. Central evaluation of MMR status was carried out using IHC with the Ventana MMR RxDx Panel (Roche Diagnostics, Rotkreuz, Switzerland).

A total of 236 patients (47.8%) had recurrent disease, and 166 (34%) of newly diagnosed cases were at primary stage IV, and 92 (18.2%) were at primary stage III.

Of this population, 23.9% had MMRd (mismatch repair deficiency) or MSI-H (microsatellite instability-high) disease, 270 (54.6%) had endometrioid carcinoma, and 15 (3%) were Asian.

The trial’s primary endpoints with a hierarchical approach were progression-free survival (PFS) in both the MMRd/MSI-H subgroup and the overall intention-to-treat (ITT) population, along with overall survival (OS) in the ITT population.

At a median follow-up, the addition of dostarlimab to chemotherapy led to a 72% reduction in the risk of disease progression or death in the MMRd/MSI-H subgroup (HR = 0.28; *p* < 0.0001). The median PFS in the MMRd/MSI-H group was not reached with dostarlimab and chemotherapy, compared to 7.7 months with chemotherapy alone. In the overall population, dostarlimab plus chemotherapy resulted in a 36% reduction in the risk of disease progression or death (HR = 0.64; *p* < 0.0001), with a median PFS of 11.8 months compared to 7.9 months in the control arm.

Grade >3 adverse events were reported in 50.6% of patients in the dostarlimab arm, compared to 46.3% in the placebo arm [6] [strength of evidence IIA].

Maintenance with dostarlimab is restricted to a maximum of 26 cycles.

Finally, Dostarlimab combined with carboplatin and paclitaxel demonstrated a statistically significant and clinically meaningful improvement in overall survival (OS) among patients with primary advanced or recurrent endometrial cancer. The combination also exhibited an acceptable safety profile [7] (strength of evidence: IIA).

##### European Medicines Agency (EMA) Registration

Dostarlimab is approved in combination with carboplatin and paclitaxel for the first-line treatment of adult patients with primary advanced or recurrent endometrial cancer who are eligible for systemic therapy.

#### 2.2.2. GY018/KEYNOTE-868 Trial (antyPD1)

The GY018/KEYNOTE-868 trial enrolled 804 patients, of whom 588 were included in the efficacy analysis, with a median follow-up period of 12 months.

Patients were randomly assigned in a 1:1 ratio to receive paclitaxel (175 mg/m^2^) plus carboplatin (AUC 5) every 3 weeks, alongside either pembrolizumab (200 mg) or placebo for six cycles. This was followed by pembrolizumab (400 mg) or placebo every 6 weeks for up to 14 additional cycles.

Mismatch repair (MMR) status and microsatellite instability (MSI) testing were centrally evaluated. Central assessment was performed using immunohistochemistry (IHC) with the VENTANA MMR IHC Panel (Ventana Medical Systems, Inc., Tucson, AZ), which includes mouse monoclonal antibodies against MLH1 (M1), PMS2 (A16-4), MSH2 (G219-1129), BRAF V600E (VE1), and a rabbit monoclonal antibody against MSH6 (SP93). Of the patients, 471 (58.6%) had recurrent disease, while 333 (41.4%) were newly diagnosed at primary stages III or IV. However, the study does not specify the distribution of newly diagnosed patients between stages III and IV.

Of this population, 225 (38.3%) had MMRd (mismatch repair deficiency) or MSI-H (microsatellite instability-high) disease, 304 (51.7%) had endometrioid carcinoma, and 31 (5.3%) were Asian. Of the Asian patients, 17 (5.8%) were assigned to the pembrolizumab arm, and 14 (4.7%) were assigned to the placebo arm.

The primary endpoint, using a direct approach with an alpha split at the outset for each cohort, was progression-free survival (PFS), assessed in both the MMRd and MMRp populations.

At a median follow-up, pembrolizumab plus chemotherapy reduced the risk of disease progression or death by 70% in the MMRd group (HR = 0.30; *p* < 0.00001). In this group, the median PFS was not reached with pembrolizumab and chemotherapy, compared to 7.6 months with chemotherapy alone. The 12-month PFS rate was 74% with pembrolizumab versus 38% in the control group. In the MMRp population, pembrolizumab plus chemotherapy also provided a significant benefit, with a median PFS of 13.1 months compared to 8.7 months in the control arm (HR = 0.54; *p* < 0.00001).

In the MMRd cohort, grade ≥3 adverse events occurred in 63.3% of patients receiving pembrolizumab and in 47.2% of those receiving placebo. In the MMRp cohort, the corresponding rates were 55.1% and 45.3%, respectively [8] [strength of evidence IIA].

Maintenance with pembrolizumab is restricted to a maximum of 14 cycles.

Data regarding OS benefits are still maturing; the observed trend is promising [9].

##### European Medicines Agency (EMA) Registration:

Pembrolizumab is approved in combination with carboplatin and paclitaxel for the first-line treatment of adult patients with primary advanced or recurrent endometrial cancer who are eligible for first-line systemic therapy.

#### 2.2.3. ATtEnd Trial

ATtEnd was an academic study conducted in patients with advanced or recurrent endometrial carcinoma/carcinosarcoma who had not previously received systemic chemotherapy for recurrence.

The study enrolled 551 patients, with a median follow-up of 28.3 months.

Patients were randomized in a 2:1 ratio to receive either atezolizumab 1200 mg or placebo, administered intravenously in combination with carboplatin (AUC 5 or 6) and paclitaxel 175 mg/m^2^, both given intravenously on day 1 of each 21-day cycle for 6 to 8 cycles. This was followed by maintenance therapy with atezolizumab 1200 mg or placebo administered intravenously every 3 weeks on day 1, continued until radiologically confirmed disease progression (as determined by the investigator), unacceptable toxicity, or withdrawal of consent. Mismatch repair (MMR) status was centrally evaluated. For central determination of MMR/MSI status, immunohistochemistry was the only method performed utilizing a panel of antibodies against MLH-1, PMS-2, MSH-2, and MHS-6 [Agilent, Santa Clara, CA, USA].

The study’s co-primary endpoints, analyzed hierarchically, were progression-free survival (PFS) in the MMRd population, as well as PFS and overall survival (OS) in the overall population.

A total of 369 patients (67.2%) had recurrent disease, and 148 (26.9%) of newly diagnosed cases were at primary stage IV, and 31 (5.7%) were at primary stage III.

Among the 549 patients in the intention-to-treat population, 125 (22.8%) had MMRd tumors, 352 (64.1%) had endometrioid carcinoma, and 112 (20.4%) were of Asian ethnicity. Of the Asian subgroup, 69 patients (19%) were assigned to the atezolizumab arm and 43 (23%) to the placebo arm. The coprimary endpoints, assessed using a hierarchical approach, were progression-free survival (PFS) in the MMRd population, and PFS and overall survival (OS) in the overall study population In the MMRd population, the addition of atezolizumab resulted in significantly improved PFS (HR 0.36; 95% CI: 0.23–0.57; *p* = 0.0005), with median PFS not reached compared to 6.9 months for the placebo arm. This PFS benefit was also observed in the overall population (HR 0.74; 95% CI: 0.61–0.91; *p* = 0.0219), with a median PFS of 10.1 months for the atezolizumab group compared to 8.9 months for the placebo group. Interim analysis of OS in all comers indicated a trend in favor of atezolizumab, but the impact on long-term survival data remains uncertain.

Grade 3 adverse events were reported in 66.9% of patients in the atezolizumab arm, compared to 63.8% in the placebo arm.

No limit was set on the number of atezolizumab maintenance cycles. [10] [strength of evidence IIA].

##### European Medicines Agency (EMA) Registration

Atezolizumab is not yet approved for the first-line treatment of adult patients with primary advanced or recurrent endometrial cancer and who are candidates for systemic therapy.

#### 2.2.4. DUO-E (antyPD-L1)

The DUO-E/GOG-3041/ENGOT-EN10 trial was a randomized, double-blind, placebo-controlled, multicenter phase III study designed to evaluate whether the addition of the anti–PD-L1 antibody durvalumab to carboplatin and paclitaxel, followed by maintenance therapy with durvalumab alone or in combination with the PARP inhibitor olaparib, could improve outcomes in patients with newly diagnosed advanced (FIGO stage III with measurable disease or stage IV based on the 2009 staging system) or recurrent epithelial endometrial cancer, excluding sarcomas [10]. [strength of evidence IIA].

The study enrolled 718 patients with advanced or recurrent epithelial endometrial cancer. Median follow-up was 12.6 months (range: 0.0–31.6) in the control arm and 15.4 months (range: 0.0–31.7) in both durvalumab-containing arms. Patients were randomized 1:1:1 to receive carboplatin (AUC 5 or 6) and paclitaxel (175 mg/m^2^) every 3 weeks for 6 cycles. The control group received a placebo during chemotherapy and as maintenance. The durvalumab group received durvalumab 1500 mg every 3 weeks during chemotherapy and every 4 weeks as maintenance, along with placebo tablets. The durvalumab–olaparib group received the same durvalumab schedule followed by maintenance with durvalumab and olaparib 300 mg twice daily until disease progression. Durvalumab arm: Platinum-based chemotherapy and paclitaxel as above, plus durvalumab 1120 mg intravenously every 3 weeks for 6 cycles, followed by durvalumab 1500 mg every 4 weeks and olaparib placebo tablets twice daily until progression.

Durvalumab–Olaparib arm: Same chemotherapy and durvalumab regimen as the durvalumab arm, with maintenance durvalumab 1500 mg every 4 weeks and olaparib 300 mg tablets twice daily until progression.

Mismatch repair (MMR) status was centrally evaluated prior to randomization. For central determination of MMR/MSI status, immunohistochemistry was the only method performed utilizing the Ventana MMR RxDx panel (Roche Diagnostics, Rotkreuz, Switzerland).

A total of 376 patients (52.4%) had recurrent disease, while 342 patients (47.6%) were newly diagnosed. Among the newly diagnosed cases, 296 (87%) were primary stage IV, and 41 (12%) were primary stage III.

Of the 718 patients in the intention-to-treat population, 143 (19.9%) had MMRd tumors, 431 (60%) had endometrioid carcinoma, and 215 (29.9%) were Asian. Of the Asian patients, 70 (29.2%) were in the Durvalumab-Olaparib arm, 72 (30.3%) were in the Durvalumab arm, and 73 (30.3%) were in the placebo arm.

The primary endpoints were progression-free survival (PFS) for both the durvalumab versus control and durvalumab–olaparib versus control comparisons. Statistically significant improvements in PFS were observed in both experimental arms: durvalumab (HR 0.71; 95% CI, 0.57–0.89; *p* = 0.003) and durvalumab–olaparib (HR 0.55; 95% CI, 0.43–0.69; *p* < 0.0001). While both regimens were superior to control, the addition of olaparib to durvalumab did not appear to confer further benefit over durvalumab alone; however, the study was not powered to directly compare the two experimental arms. These findings support the integration of immunotherapy into first-line chemotherapy and suggest a potential role for PARP inhibition, though its impact on long-term survival remains to be determined. Grade 3 or greater adverse events were reported in 230 of 430 patients (55.6%) in the durvalumab/olaparib arm, 159 of 418 patients (38%) in the durvalumab arm, and 158 of 405 patients (39.0%) in the placebo arm.

No limit was set on the number of maintenance cycles. [11] (strength of evidence: IIA).

##### European Medicines Agency (EMA) Registration

Durvalumab in combination with carboplatin and paclitaxel is indicated for the first-line treatment of adults with primary advanced or recurrent endometrial cancer who are candidates for systemic therapy, followed by maintenance treatment with: Durvalumab as monotherapy in endometrial cancer that is mismatch repair deficient (MMRd), Durvalumab in combination with olaparib in endometrial cancer that is mismatch repair proficient (MMRp).

A summary of randomized controlled trials evaluating first-line systemic treatments for endometrial cancer is presented in Table 2.

#### 2.2.5. Review of Phase III Trials on Immunotherapy

A meta-analysis by Bogni et al. evaluated phase III trials on immunotherapy for endometrial cancer, examining its effectiveness in the general population as well as in different subtypes (MMRd and MMRp) using statistical tools from the R Project. Pooled results from the RUBY, AtTEnd, and DUOE trials indicated that immunotherapy improved progression-free survival across all patients (HR 0.70, 95% CI 0.62–0.79). In the NRG-GY018 trial, combining chemotherapy with immunotherapy resulted in a longer median progression-free survival (PFS) compared to chemotherapy plus placebo (18.8 vs. 8.5 months). In the MMRd/MSI-H subgroup, the combination significantly improved PFS (HR 0.33; 95% CI, 0.23–0.43), while a more modest but still significant benefit was observed in the MMRp/MSS subgroup (HR 0.74; 95% CI, 0.60–0.91) The same trials also showed improved overall survival with immunotherapy in all patients (HR 0.75, 95% CI 0.63–0.89). In the MMRd/MSI-H subgroup, immunotherapy was associated with better survival (HR 0.37, 95% CI 0.22–0.61), but no survival benefit was found in the MMRp/MSS group (HR 0.86, 95% CI 0.63–1.17) [12] [Strength of evidence IA].

## 3. How Does New Evidence Contribute to Updates in Treatment Guidelines?

The most recent randomized controlled trials (RCTs) primarily influence recommendations for first-line systemic treatment. These updates apply to locally advanced endometrial cancer with residual disease after surgery (incompletely resected FIGO stage III-IVA), metastatic disease (FIGO stage IVB), and unresectable recurrence.

Consequently, the previously established diagnostic and treatment guidelines for clinico-radiological FIGO stage I/II (operable/inoperable early endometrial carcinoma) and stage IIIA/B/C/IVA (operable/inoperable locally advanced endometrial carcinoma) remain unchanged (Recommendation 2023v1) [1], except for a single modification regarding the adjuvant treatment of FIGO I/II R0 carcinosarcoma cases.

Although there is no robust evidence, the expert panel recommends considering the addition of chemotherapy to radiotherapy (brachytherapy + external beam radiotherapy) as adjuvant treatment for FIGO I/II R0 carcinosarcoma cases. [expert opinion] [strength of evidence V] (grade of recommendation: 1).

**Figure 2:**
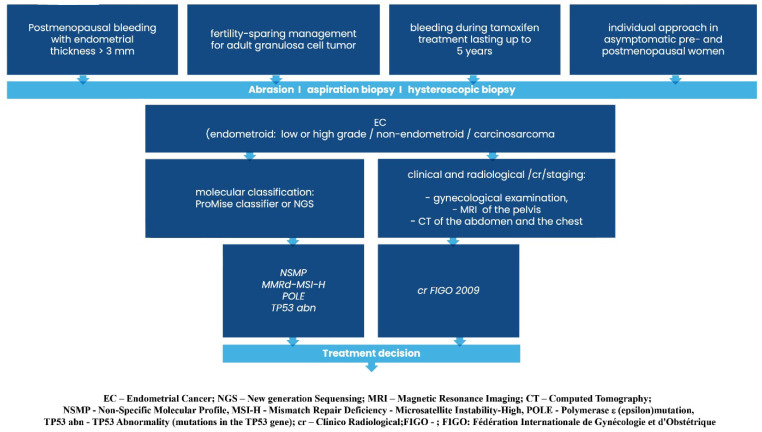
Diagnosis prior to treatment decision.

**Figure 3:**
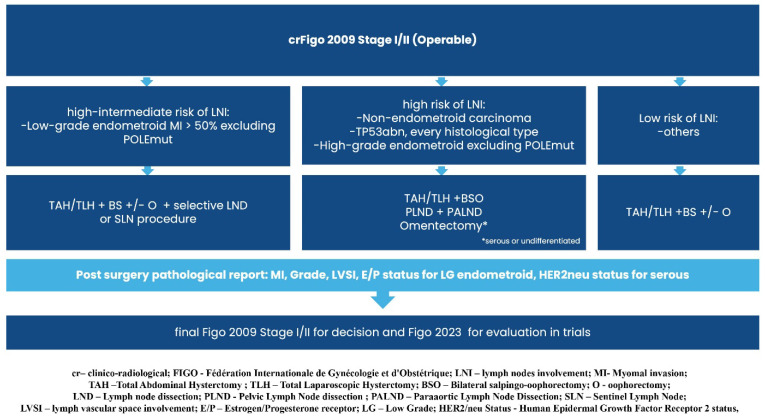
Treatment of clinical and radiological FIGO stage I/II (Operable).

**Figure 4:**
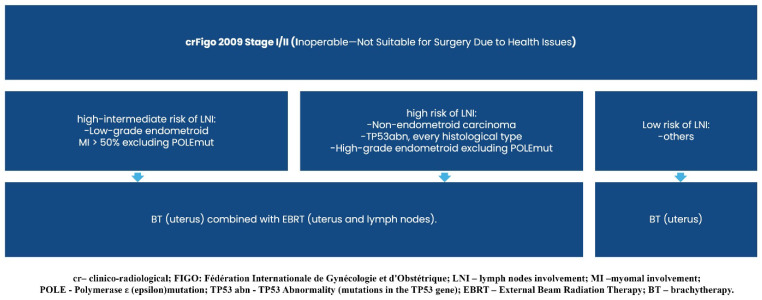
Treatment of clinical and radiological FIGO stage I/II (inoperable).

**Figure 5:**
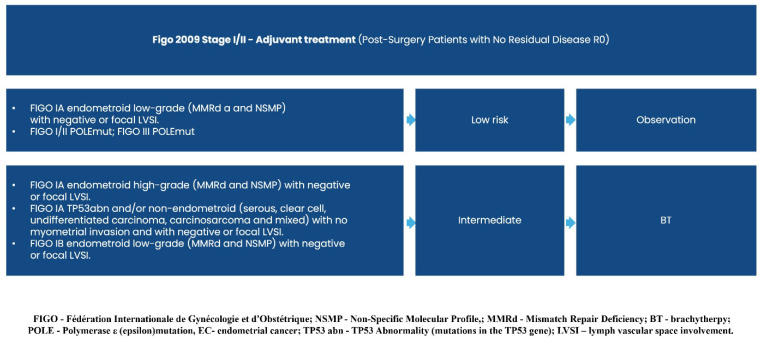
Adjuvant Treatment (Post-Surgery Patients, FIGO I/II without Residual Disease R0)—cases with low and intermediate risk of recurrence.

**Figure 6:**
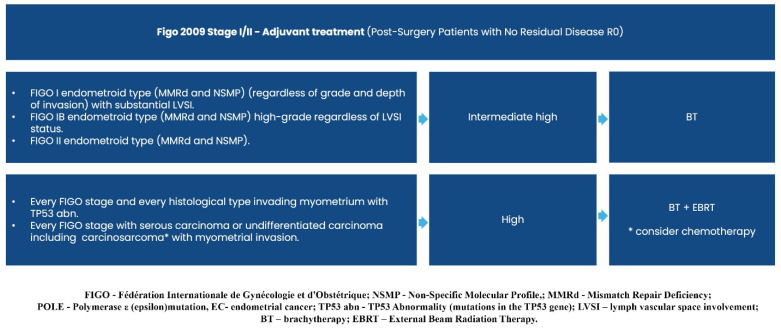
Adjuvant Treatment (Post-Surgery Patients FIGO I/II without Residual Disease R0)—cases with intermediate, high, and high risk of recurrence.

**Figure 7:**
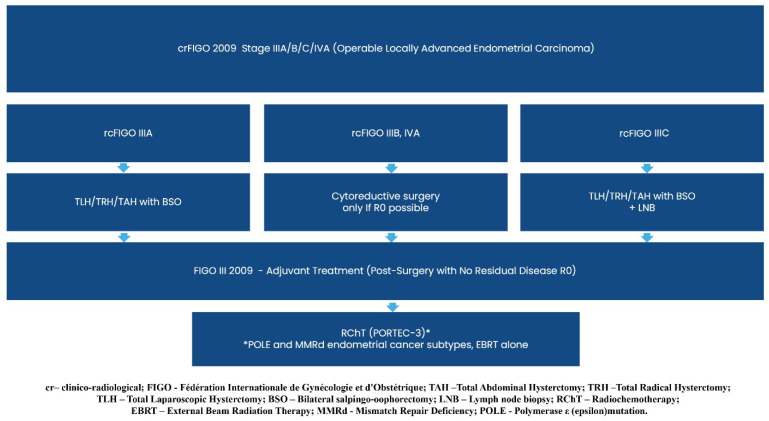
Treatment of clinical and radiological FIGO Stage IIIA/B/C/IVA (Operable Locally Advanced Endometrial Carcinoma).

**Figure 8:**
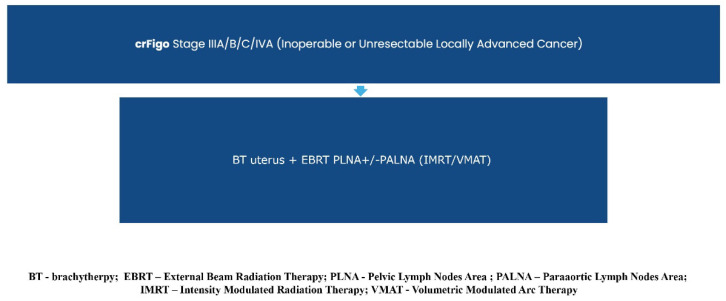
Treatment of Clinical and Radiological FIGO Stage IIIA/B/C/IVA (inoperable Locally Advanced Endometrial Carcinoma).

Graphical presentations illustrating the recommended management of endometrial cancer patients, excluding those requiring systemic treatment, can be found in Figure 2, Figure 3, Figure 4, Figure 5, Figure 6, Figure 7 and Figure 8. The evidence supporting these decisions is provided in the main text of the previous Recommendation 2023v1 [1].

### 3.1. First Line Systemic Treatment Changes

Before the publication of recent data, the following first-line systemic treatments were recommended:

In cases of low-grade endometrioid carcinoma expressing progesterone (PR) and estrogen (ER) receptors, hormone therapy was the treatment of choice [13,14] (strength of evidence IIIA; grade of recommendation 2A).

In cases of high-grade endometrioid and non-endometrioid carcinoma types (serous, clear cell, carcinosarcoma), carboplatin plus paclitaxel was the preferred regimen [15] (strength of evidence IIA; grade of recommendation 1).

In cases of HER2-positive serous carcinoma, the addition of trastuzumab to carboplatin plus paclitaxel was recommended as it was proven that it prolongs progression-free survival/PFS/ [16] (strength of evidence IIA; grade of recommendation 1). Furthermore, recent evidence confirmed that this treatment approach increases overall survival (OS) [17].

Recent evidence has led to significant updates for first-line systemic treatment, as outlined below:

In cases of low-grade endometrioid carcinoma expressing progesterone (PR) and estrogen (ER) receptors, hormone therapy remains the treatment of choice [13,14] (strength of evidence IIIA; grade of recommendation 2A).

In cases of high-grade endometrioid and non-endometrioid carcinoma types (serous, clear cell, carcinosarcoma), the initial treatment should now be immunotherapy (I/O) plus chemotherapy (CHTH) followed by I/O maintenance therapy instead of chemotherapy (CHTH) alone [6,8,10,11] [strength of evidence IIA, IIA, IIA, IIA] (grade of recommendation 1).

**Recommendation:**

Initial Treatment: I/O plus CHTH.

Administer one of the available PD-1 or PD-L1 inhibitors: dostarlimab, pembrolizumab, or Durvalumab in combination with carboplatin and paclitaxel every 3 weeks for the first 6 to 8 cycles.

Maintenance Therapy:

Continue I/O as monotherapy or in combination, based on the agent used:

Dostarlimab: Administer every 6 weeks until disease progression or unacceptable toxicity, but no longer than 26 cycles.

Pembrolizumab: Administer every 6 weeks until disease progression or unacceptable toxicity, but no longer than 14 cycles.

Durvalumab: Administer every 4 weeks, either as monotherapy in endometrial cancer that is mismatch repair deficient (MMRd) or in combination with Olaparib in endometrial cancer that is mismatch repair proficient (MMRp)*, until disease progression or unacceptable toxicity.

**Figure 9:**
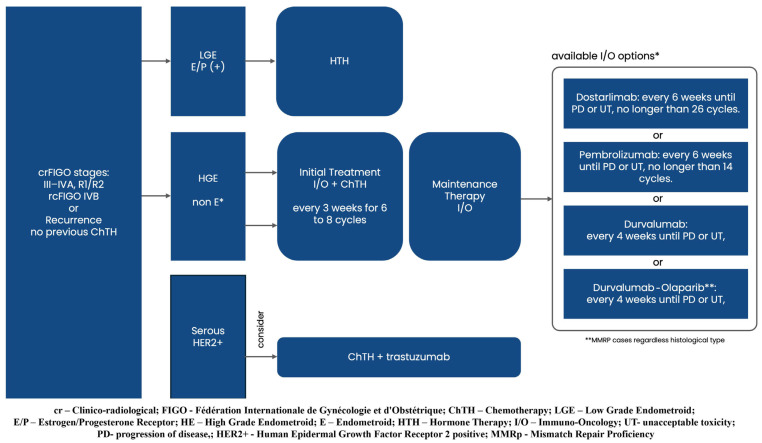
First line systemic treatment: Locally Advanced Cancer cases with residual disease after surgery (incompletely resected FIGO III-IVA), Metastatic Disease (Note: M1—FIGO IVB: presence of metastases outside the pelvis or in the nonregional lymph node), and Recurrence.

*For HER2-positive, MMRp serous carcinoma cases, the addition of trastuzumab to carboplatin and paclitaxel is recommended for consideration [expert opinion; strength of evidence IIA] (Figure 9).

### 3.2. Second-Line Systemic Treatment Changes

#### 3.2.1. For Patients Who Progressed After at Least One Cycle of Platinum-Based Chemotherapy and Did Not Receive I/O Plus CHTH During Initial Treatment, the Recommendations Remain Unchanged from 2023v1 as Outlined Below

For patients with mismatch repair deficiency MMRd/MSI-H, the treatment of choice is a PD-1 inhibitor such as dostarlimab or pembrolizumab, or a combination of a PD-1 inhibitor (pembrolizumab) and lenvatinib (grade of recommendation 1).

Immunotherapy is the treatment of choice for advanced or recurrent endometrial cancer [18,19,20] [strength of evidence: IIA, IIC, IID] (grade of recommendation: 1).

For patients with MMRd/MSI-H tumors, recommended options include PD-1 inhibitors such as dostarlimab or pembrolizumab, or the combination of pembrolizumab with lenvatinib (grade of recommendation: 1). However, due to the high rate of treatment discontinuation associated with the pembrolizumab–lenvatinib combination, PD-1 inhibitor monotherapy is preferred in this subgroup [expert opinion; strength of evidence: V] (grade of recommendation: 2B).

For patients with MMRp tumors, the preferred treatment—where available—is the combination of pembrolizumab and lenvatinib [strength of evidence: IIA] (grade of recommendation: 1).

Chemotherapy with carboplatin and paclitaxel is recommended for second-line treatment only for carcinosarcoma cases.

#### 3.2.2. For Patients Who Received I/O Plus Chemotherapy as Initial Treatment, the Options for Second-Line Systemic Therapy Remain Unclear and Are Currently Determined by the Treating Physician’s Discretion

**Figure 10:**
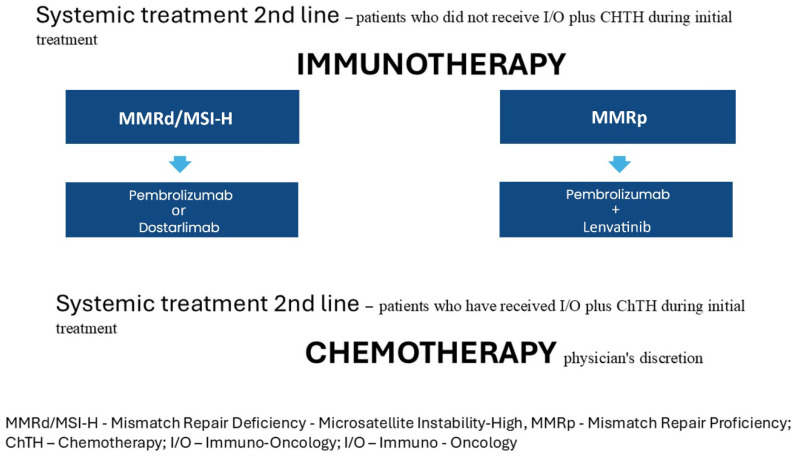
Second-line systemic treatment.

Second Line Systemic Treatment Strategies Are Summarized in Figure 10.

## 4. Conclusions: Key Changes from 2023v1 to 2025v1

High-grade endometrioid and non-endometrioid carcinomas (all molecular types):

Initial therapy for all patients now includes I/O plus CHTH, followed by maintenance, compared to CHTH alone in 2023v1.

MMRp positive cases could benefit from the addition of Olaparib to I/O (Durvalumab) plus CHTH, which is newly emphasized in 2024v1.

HER2-positive MMRp serous carcinoma cases could benefit from the addition of trastuzumab to CHTH, which was previously emphasized in 2023v1.

Second-line treatment:

Recommendations for patients who did not receive I/O plus CHTH during initial treatment remain unchanged.

For patients who received I/O plus CHTH, second-line options are yet to be established.

## Figures and Tables

**Table 1:** Comparison of FIGO 2009 and FIGO 2023 staging systems for endometrial carcinoma [4].

FIGO 2009 Stage I	FIGO 2023 Stage I
Tumor is limited to the uterine corpus.	A combination of the following features: histological typea, presence and depth of myometrial invasion (inner versus outer half), and absent or focal LVSI ^b^
Subclassified as stage IA, indicating no myometrial invasion or invasion of less than 50% of the myometrial thickness, and stage IB, indicating myometrial invasion equal to or greater than 50%.	Classification into stage IA or IB now applies exclusively to non-aggressive histological subtypes ^a^ with absent or focal LVSI ^b^
	For non-aggressive histological types ^a^ with absent or focal LVSI ^b^, the updated classification reintroduces a distinction between tumors confined to the endometrium (now classified as IA1), those with less than 50% myometrial invasion (IA2), and those with invasion equal to or exceeding 50% (IB).
	For aggressive histological types ^a^, the updated system introduces stage IC for tumors without myometrial invasion and classifies any degree of myometrial invasion as stage IIC.
	Ovarian involvement is now permitted within staging criteria if all of the following conditions are met: low-grade endometrioid histology; absent or superficial myometrial invasion (<50%); absent or focal LVSI ^b^; no evidence of additional metastatic disease; unilateral ovarian involvement confined to the ovary, without capsular invasion or rupture.
**FIGO 2009 Stage II**	**FIGO 2023 Stage II**
Tumor confined to the uterus with extension into the cervical stroma	A combination of tumor features—including cervical stromal invasion, substantial LVSI ^b^, and myometrial infiltration by an aggressive histological subtype ^a^.
	IIA—a non-aggressive histological type ^a^ with extension to
	IIB—a non-aggressive histological type ^a^ with substantial LVSI ^b^
	IIC—an aggressive histological type with any degree of myometrial invasion.
**FIGO 2009 Stage III**	**FIGO 2023 Stage III**
Local and/or regional spread outside of the uterus excluding bladder/intestinal lining, and distant sites.	Local and/or regional tumor spread.
Tubo-ovarian and serosal involvement are grouped under stage IIIA.	Stage IIIA is now subdivided into:
	IIIA1, Tubo-ovarian involvement
	IIIA2, Subserosal and serosal involvement The concept of the uterine subserosa as a distinct anatomical site has been introduced.
Vaginal and parametrial tumor involvement are grouped under stage IIIB.	Stage IIIB is subdivided into IIIB1 indicating vaginal and/or parametrial tumor involvement IIIB2 indicating pelvic peritoneal involvement).
Nodal micro- and macrometastasis are grouped under stage IIIC1 (pelvic) and IIIC2 (para-aortic).	Stage IIIC1, indicating pelvic lymph node involvement, is now subdivided into IIIC1 for micrometastases and IIIC1 for macrometastases. Similarly, Stage IIIC2, indicating para-aortic lymph node involvement, is subdivided into IIIC2 for micrometastases and IIIC2 for macrometastases
**FIGO 2009 Stage IV**	**FIGO 2023 Stage IV**
Stage IVB includes abdominal peritoneal spread as well as distant metastases to the lungs, liver, brain, bone, and non-regional lymph nodes (the inguinal region or above the renal vessels).	Abdominal peritoneal spread is classified as stage IVB. Distant metastases to the lungs, liver, brain, bone, and non-regional lymph nodes (the inguinal region or above the renal vessels) are grouped as stage IVC
In FIGO 2023 staging for stages I and II, POLE-mutated tumors are designated as IAm POLEmut and p53-abnormal tumors as IICm p53abn, irrespective of anatomical extent, degree of LVSI, or histological subtype. Tumors with no specific molecular profile (NSMP) or mismatch repair deficiency (MMRd) do not influence staging	
^a^ Histological Classification: **Non-aggressive histological types:** FIGO grade 1 and 2 endometrioid carcinomas. **Aggressive histological types:** FIGO grade 3 endometrioid, serous, clear cell, undifferentiated, dedifferentiated, mesonephric-like, gastrointestinal-type mucinous carcinomas, and carcinosarcomas.	
^b^ Lymphovascular space invasion (LVSI): Substantial: ≥5 vessels involved. Focal: <5 vessels involved.	

FIGO—International Federation of Gynecology and Obstetrics; MMRd—Mismatch Repair Deficiency.

**Table 2:** Comparison of Randomized Controlled Trials on First-Line Systemic Treatments for Endometrial Cancer.

RCT	Antibody	Cohort	Randomization Strategy	Patient Population		Duration	PFS Benefit			Grade3 Adverse Events IO vs. Control
				Recurrent Disease	Newly Diagnosed Stage III/Stage IV		Overall Population	MMRd	MMRp	
RUBY/ENGOT-EN6/GOG3031	antyPD-1	494	1:1 A: CT+ Dostarlimab B: CT	239 (47.7%)	258 (52.3%)	up to progression max. 36 months	HR 0.64 (95% CI 0.51–0.80) *p* < 0.001	HR 0.28 (95% CI 0.16–0.50) *p* < 0.0001	HR 0.76 (95% CI 0.59–0.98)	174 (72.2%) vs. 148 (60.2%)
NRG-GY018	antyPD1	813	1:1 A: CT+ Pembrolizumab B: CT	not recorded	not recorded	up to progression max. 25.5 months	HR 0.30 (95% CI, 0.19–0.48), *p* < 0.001	HR 0.30 (95% CI 0.19–0.48) *p* < 0.0001	HR 0.54 (95% CI 0.41–0.71)	MMRd 69 (63.3%) vs. MMRp 50 (47.2%) vs. 124 (45.3%)
AtTEnd	antyPDL1	549	2:1 A: CT+ Atezolizumab B: CT	243 (67.5%) vs. 126 (66.7%)	117 (32,5%) vs. 62 (33,3%)	up to progression	HR 0.74 (95% CI 0.61–0.91) *p<* 0.003	HR 0.38 (95%CI 0.23–0.57) *p<* 0.0005	HR 0.92 (95% CI 0.73–1.16)	No data
DUO-E	antyPDL1	479	1:1:1 A: CT B: CT + Durvalumab C: CT + Durvalumab +olaparib	125 vs. 126	116 vs.114	up to progression	HR 0.71 (95% CI 0.57–0.89), *p* < 0.0219	HR 0.42 (95%CI 0.22–0.80)	HR 0.77 (95% CI 0.60–0.97) *p* < 0.0001	No data
DUO-E	antyPDL1 + Olaparib	480	1:1:1 A: CT B: CT + Durvalumab **C:** CT + durvalumab +olaparib	125 vs. 126	114 vs. 116	up to progression	HR:0.55 (95% CI 0.43–0.69); *p* < 0.0001	HR:0.28 (95%CI, 0.10–0.68)	No data	No data

PFS—progression-free survival; MMRd—mismatch repair deficiency; MMRp—mismatch repair proficiency; IO—Immuno Oncology.
